# Presence of neutrophil extracellular traps (NETs) in different types of human urinary tract infections (UTI). A pilot study

**DOI:** 10.3389/fimmu.2026.1745166

**Published:** 2026-03-16

**Authors:** Lena Schröder Alvarez, Iván Conejeros, Gabriel Espinosa, Constanza Salinas-Varas, Benjamin Ott, Markus Weigel, Can Imirzalioglu, Moritz Fritzenwanker, Anita C. Windhorst, Torsten Hain, Anja Taubert, Carlos Hermosilla, Florian Wagenlehner

**Affiliations:** 1 Institute of Parasitology, Justus Liebig University Giessen, Giessen, Germany; 2 Clinic of Urology, Pediatric Urology and Andrology, Justus Liebig University Giessen, Giessen, Germany; 3 Institute of Medical Microbiology, Medical Microbiome-Metagenome Unit (M3U), Justus Liebig University Giessen, Giessen, Germany; 4 German Center for Infection Research (DZIF), Partner Site Giessen-Marburg-Langen, Justus Liebig University Giessen, Giessen, Germany; 5 Institute of Medical Informatics, Justus Liebig University Giessen, Giessen, Germany

**Keywords:** microbiome, NETs, neutrophils, PMN, UTI

## Abstract

**Introduction:**

Activated polymorphonuclear neutrophils (PMN) release neutrophil extracellular traps (NETs) composed of a web-like DNA core, concomitant with nuclear histones, granular peptides and enzymes. NETs in human urine and their potential role in human urinary tract infections (UTI) pathogenesis is still understudied. This pilot study aimed to analyze presence of NETs in urine samples of patients with different types of UTI.

**Methods:**

Urine and blood samples were collected from three cohorts: group (A) included females (*n* = 24) with cystitis (*n* = 10), pyelonephritis (*n* = 6), and asymptomatic bacteriuria (*n* = 8); group (B) composed of males with catheter-associated UTI (*n* = 20) and a control group (C) consisting of healthy patients of mixed gender (*n* = 20). NETs in urine samples were confirmed by immunofluorescence-based detection of neutrophil elastase and citrullinated histone. The presence of granular enzymes (myeloperoxidase, cathelicidin), calprotectin (subunits S100A8, S100A9) and CD15^+^ PMN were detected by ELISA, western blot and flow cytometry, respectively. To study potential associations of NETs with the respective UTI microbiome, bacterial spectrum of each urine sample was estimated by 16S rRNA gene analysis.

**Results and discussion:**

On average, 23.29% ± 16.89% of PMN forming NETs were detected in group A [subgroups cystitis (27.72% ± 17.88%), pyelonephritis (22.75% ± 12.91%), asymptomatic bacteriuria (18.17% ± 17.14%)] and 30.63% ± 17.88% in group B, with no differences observed between UTI groups, including patients with asymptomatic bacteriuria. For the control group (group C), a low incidence of NET-releasing cells was observed (0.32% ± 1.42%), resulting in a significant difference (*p* < 0.05) when compared to all UTI groups studied. Furthermore, different NET-phenotypes [i. e. spread NETs (*spr*NETs), diffuse NETs (*diff*NETs) and aggregated NETs (*agg*NETs)] were detected in both UTI groups. The presence of NET-associated proteins was confirmed in all UTI groups, but absent in the control samples. Microbiome analyses revealed a reduced microbial variability within UTI samples with the predominance of the bacterial family *Enterobacteriaceae*. Overall, PMN-derived NETs were consistently found in all UTI samples, suggesting a role of NETs in diverse UTI pathologies. Future studies should investigate its utility as an inflammatory biomarker in clinical human UTI.

## 1 Introduction

Within professional phagocytes of the innate immune system, polymorphonuclear neutrophils (PMN) play a central role in early defense via phagocytosis, reactive oxygen species (ROS) generation, cytokine/chemokine production, release of extracellular vesicles (EV), degranulation of antimicrobial peptides, and the formation of neutrophil extracellular traps (NETs) (1–3). The process of NET formation, known as NETosis (4), involves the release of web-like DNA into the extracellular space, where it binds microbicidal effector proteins derived from the nucleus, cytosol, or granules (5). Mammalian-derived NETs can occur in various phenotypes, such as spread NETs (*spr*NETs), diffuse NETs (*diff*NETs), aggregated NETs (*agg*NETs), cell-free NETs and anchored NETs (6–9). Irrespective of phenotypes, NETs can efficiently trap and eventually kill pathogens (10). NET release involves arginine deiminase 4 (PAD4)-mediated histone citrullination facilitating nuclear chromatin decondensation, actin cytoskeleton and microtubules disassembly and remodelling, as well as the release of extracellular vesicles (11). The disintegration of intracellular membranes allows enzymes like neutrophil elastase (NE) and myeloperoxidase (MPO) to interact, to translocate to the nucleus and to attach to chromatin (12, 13). NET-associated molecules include the antibacterial and cytotoxic protein cathelicidin (LL-37), released from peroxidase-negative PMN granules (14, 15), and the cytosolic antifungal and antibacterial heterodimer calprotectin, composed of S100A8 and S100A9 subunits (12). Interestingly, elevated calprotectin levels have been reported in extracellular fluids of patients with inflammatory diseases, including vasculitis and urinary tract infections (UTI), where calprotectin may serve as an inflammatory effector molecule (12, 16).

Human PMN can rapidly migrate into the urinary tract in response to UTI (17), however, the exact mechanism of PMN extravasation or their entry point into the urinary tract still remain unclear (18). Elevated leukocyte numbers in the urine (i. e. leukocyturia) along with nitrite detection serve as classical clinical markers for diagnosing UTI (19). These are commonly detected by urine dipstick tests, a rapid method for UTI diagnosis, which measures leukocyte esterase, a marker for the presence of active leukocytes (18). Most human UTI cases are of bacterial origin, with uropathogenic *Escherichia coli* being the most common species (20). Human UTI can manifest as mild infections, with cystitis being the most common type (18), which predominantly affects women (21). Even though cystitis-related inflammatory reactions are typically limited to the bladder, it can ascend to the renal pelvis leading to pyelonephritis and eventually urosepsis, which is potentially life-threatening (18). Another entity is the situation of asymptomatic bacteriuria, lacking any signs of a UTI, but presenting bacteria and leukocytes in the urine. Furthermore, in intensive care units, where intubated patients cannot express symptom-related complaints, biomarkers enabling UTI differentiation would be highly relevant to recognize infections as soon as possible, whereby new clinical markers can aid for either rapid and/or ‘point of care’ diagnostics (22). In this context, NET formation, a reaction of PMN to microbial presence (23), has been proposed as an useful clinical inflammatory biomarker, however, its precise role in the pathogenesis of UTI warrants further investigation since uncontrolled NETosis may also result in inflammation-driven damage (18). Notably, a link between exacerbating NET formation and UTI has recently been proposed (23–25). Consistently, elevated levels of NET-related components in the urine [LL-37, MPO and extracellular DNA (exDNA)] correlated with leukocyturia in pediatric pyelonephritis (23). Additionally, murine NET formation correlated with higher bacterial loads, implying that NET formation is not merely a consequence of UTI but also plays a role in immunological control of UTI infections (23). Accordingly, Goldspink et al. (2023) recently demonstrated that drugs like furosemide decrease NET formation by reducing the sodium chloride gradient in the kidney, but thereby enhancing the severity of pyelonephritis. In line, the virulence factor TcpC of uropathogenic *E. coli* indeed aggravated kidney infection by suppressing extrusion of NETs (25).

The current study investigates the occurrence of NETs across different UTI types and its association to the urinary microbiome, using human *ex vivo* body fluids (i. e. blood and urine). Therefore, women with a localized infection of the bladder (i. e. cystitis), an invasive infection of the kidney (pyelonephritis) or lacking symptoms but showing bacteriuria (i. e. asymptomatic bacteriuria), as well as male patients with biofilm forming UTI [catheter-associated UTI (CA-UTI)] have been included. NETs were microscopically identified, categorized into phenotypes (*spr*NETs, *diff*NETs, *agg*NETs) and quantified by immunofluorescence microscopy (IFM) by the detection of DNA, NE and citrullinated histones (cit.H). The cell surface marker CD15 (26) was used to detect human PMN in flow cytometry, while the presence of MPO, LL-37 and calprotectin was analyzed by ELISA and Western blotting (WB). Additionally, 16S rRNA gene microbiome analysis was performed on single urine samples to characterize the individual pathogenic spectrum.

## 2 Materials and methods

### 2.1 Ethics statement

Donors of urine and blood samples were informed of the study protocol and their privacy was guaranteed. All participants signed a written informed consent form before participating in this work agreeing that their samples would be exclusively used for scientific purposes. This study was conducted following the approval of the Ethic Committee of the Justus Liebig University Giessen, Giessen, Germany (AZ 280/20) and in accordance to the Declaration of Helsinki.

### 2.2 Study design and sample collection

A total of 71 patients (*n* = 71) were enrolled and divided into three cohorts: women with UTI (group A), men with UTI (group B) and a mixed-gender control group (group C). Patients in group A and B presented at the University Hospital Giessen between October 2023 and April 2024. Inclusion criteria for patients with UTI were limited to adult individuals over the age of 18, non-pregnant, without prior antibiotic treatment, and presence of leukocyturia. Control group participants were healthy individuals without urological history, and no leukocyturia. Besides sample collection of urine and blood, all participants provided informed consent and signed a privacy declaration, with their medical history recorded via a detailed questionnaire. The questionnaire collected demographic data (age, gender), information on current medication, previous antibiotic use and medical history including risk factors for UTI (19). The questionnaire further documented the onset of pain and specific UTI-related symptoms, including dysuria, haematuria, costovertebral angle tenderness, fever, suprapubic pain, chills, and urinary urgency (19). Further, participants were questioned about their urological history, specifically inquiring whether they had a prior history of UTI or the presence of any foreign material, such as a catheter or ureteral stent.

Samples were processed following hospital routine procedures and analysed by the Departments of Clinical Chemistry and Microbiology at the Justus Liebig University Giessen. Diagnostic classification was based on the score developed by Bilsen et al. (2024), which is a consensus standardized diagnostic tool designed to assess the likelihood of UTI. It incorporates clinical localized or systemic symptoms, host inflammatory signals (e. g. pyuria) and microbiological results. Each category is assigned a score from 0 to 3 points. Scores ≥ 8 points indicated a definite UTI, 5–7 probable, 3–4 possible, and < 3 unlikely (22). UTI groups were further classified according to EAU guidelines on urological infections (19) by correlating questionnaire-based symptom data with diagnoses from medical records. Group A (women with UTI) was subdivided into three clinical categories: cystitis (*n* = 10), pyelonephritis (*n* = 6) and asymptomatic bacteriuria (*n* = 8). Cystitis was defined by symptoms like dysuria, enhanced urinary frequency and urgency, with the absence of vaginal discharge. Pyelonephritis was identified by systemic signs including fever, chills, flank pain, nausea, vomiting, with or without accompanying lower urinary tract symptoms. Asymptomatic bacteriuria was defined as the presence of significant bacteriuria in the absence of urinary tract symptoms (19). In contrast, group B (men with UTI) was clinically uniform, consisting exclusively of patients with CA-UTI, representing a typical biofilm-associated infection. CA-UTI was defined by the presence of a urinary catheter at the time of sampling or within the previous 48 h. Diagnostic criteria obligatorily included systemic signs like fever, chills, altered mental status, in addition to local symptoms, such as flank pain, haematuria, pelvic discomfort, or dysuria (19). Of note, almost all patients in this group were asymptomatic and presented solely for routine catheter replacement.

For sample collection, blood and urine were obtained from all patients. Blood was collected to monitor systemic inflammation and was received by venous puncture in EDTA containing K3E tubes (9 mL/02.1066.001, Sarstedt). Human PMN were isolated from whole blood by magnetic negative selection using the EasySep™ Direct Human Neutrophil Isolation Kit (#19666, STEMCELL) by following the manufacturer’s protocol. Then, PMN were pelleted (800 x *g*, 8 min), resuspended in 5 mL sterile RPMI 1640 (Sigma-Aldrich) and counted in a Neubauer chamber. Isolated PMN served as internal controls in IFM for testing antibodies stains to detect NETs and were also used as positive controls in WB analysis. Moreover, fresh urine was collected via a sterile bladder catheter or by mid-stream urine (control group) into a sterile urine container (Med Comfort^®^, L-09184-S). A total of 10 mL urine was centrifuged at 600 x *g* for 10 min at room temperature (RT). After removing the supernatant, the cell pellet was diluted 1000:1 with Sytox Green^®^ (#S7020, Thermo Fisher Scientific) and mounted in a glass slide. An inverted IX81^®^ epifluorescence microscope equipped with an XM 10^®^ digital camera (both Olympus) was used for image acquisition.

### 2.3 NET detection in urine by immunofluorescence microscopy

1 mL of urine cell suspension was added to a poly-_L_-lysin (0.001%, Sigma Aldrich) pretreated coverslip (15 mm diameter, Thermo Fisher Scientific) and centrifuged (211 x *g*, 5 min) at RT. Samples were fixed with 4% PFA (Merck) and stored at 4 °C until further use.

IFM-based analysis of NET-associated proteins was performed by using anti-histone H4 Arg3 citrullination (1:100, cit.H, rabbit, Millipore 07-596) and anti-neutrophil elastase (1:200, NE, mouse, Abcam ab254178) antibodies (**Table 1**). Therefore, samples were first blocked in 750 µL permeabilization solution [sterile PBS 1X/3% bovine serum albumin (BSA; #A7906-100, Sigma-Aldrich, Darmstadt, Germany)/0.3% Triton X-100 (#T87878, Sigma Aldrich)] for 60 min at RT and probed with primary antibodies overnight in permeabilization solution at RT. After three washes with sterile PBS, samples were incubated in secondary antibody solutions (**Table 1**) for 30 min at RT in the dark. Coverslips were mounted with a drop of anti-fading buffer Fluoromont G^®^ with DAPI (Thermo Fisher Scientific, Braunschweig, Germany, 495952). While protected from direct sunlight, coverslips dried for at least 24 h at RT before microscopic analysis. For image acquisition a BZ-X800 microscope (Keyence) was used, thereby applying identical brightness and contrast conditions within the datasets of each experiment. Five randomly chosen images were acquired at 20x magnification in three channels [i. e. DAPI (blue), NE (red) and citrullinated histone (green)] to obtain a representative overview of each sample. To determine the total cell number, cells were counted manually in each sample in the DAPI channel using the FIJI/Image J^®^ counting tool. The proportion of human PMN in each sample was calculated by dividing the number of PMN in the NE channel by the total cell number in the DAPI channel.

**Table 1:** Primary and secondary antibodies used for immunofluorescence staining.

Antigen	Dilution	Origin	Company cat. no.	Secondary antibody	Dilution
Neutrophil Elastase	1:200	Mouse	Abcam ab254178	Alexa Fluor 594 A11005	1:500
Histone H4 Arg3 citrullination (cit.H)	1:100	Rabbit	Millipore 07-596	Alexa Fluor 488 A11008	1:500

NET phenotypes were microscopically identified in image merges, where all NET components (DNA, NE and cit.H) were visible. The percentage of NETs was calculated as [number of NET-positive PMN/total PMN] x 100 (27, 28) in five randomly taken images per sample. An average NET percentage was calculated and used for group comparison.

### 2.4 Quantification of LL-37 and MPO in human urine via ELISA

LL-37 and MPO were detected in human urine samples by commercial ELISAs [LL-37 Human Elisa kit, Hycult Biotech (#HK321); Human Myeloperoxidase DuoSet Elisa Kit, R&D Systems (DY3174)]. Both ELISAs were performed according to the manufacturer’s instructions as reported elsewhere (23). Fresh urine samples were centrifuged (1500 x *g*, 15 min, RT), supernatants were collected into fresh sterile Eppendorf tubes and diluted in dilution buffer (1:1 for LL-37 detection for all groups; or 1:4000 and 1:100 for MPO for group A/B and group C, respectively). For the MPO-specific ELISA kit, a 96-well high-binding polystyrene microplate (F-bottom, Greiner Bio-One, #655061) was used.

The samples were analyzed by a Varioskan Flash Reader^®^ (Thermo Fisher Scientific, MA, USA) by 450 nm wavelength readings (and additionally at 570 nm wavelengths for MPO ELISA). Standard curves and sample concentrations of LL-37 and MPO were calculated using GraphPad PRISM^®^ V10.3.0 software package (GraphPad software, USA) according to the manufacturer instructions.

### 2.5 Calprotectin (subunits S100A8 and S100A9) detection in urine via Western blot analysis

Fresh urine samples (0.5 mL) were transferred to a sterile Eppendorf tube, centrifuged three times (211 x *g*, 5 min, RT) and washed in between with 500 µL sterile PBS. In parallel, peripheral blood PMN were isolated (section 2.2), serving as positive controls. Blood PMN were centrifuged twice at 1500 rpm for 5 min and resuspended in between in 1 mL sterile PBS. Urine samples and blood PMN were treated 1:1 with RIPA buffer (50 mM Tris-HCl, pH 7.4; 1% NP-40; 0.5% Na-deoxycholate; 0.1% SDS; 150 mM NaCl; 2 mM EDTA; 50 mM NaF; all Roth, Karlsruhe, Germany) supplemented with 5 µL protease inhibitor cocktail (#P8340, Sigma-Aldrich, 1:200), 10 µL orthovanadate (#ab120386, Abcam, 1 mM) and 5 µL PMSF (#ab141032, Abcam, 1 mM). Samples were stored at -20 °C (urine) and -80 °C (blood) until further analysis.

For protein isolation, thawed samples were sonicated by ultrasound (Bandelin sonorex, Berlin, Germany 20 s, 5 cycles). After centrifugation (4 °C, 15300 x *g*, 10 min, Eppendorf 5430R), the protein concentration in supernatants was determined by the absorbance at 280 nm, using µDrop Plates^®^ (#N12391 for Varioskan Flash^®^; both Thermo Scientific). The protein concentration of the samples was calculated relative to the provided standard.

For electrophoresis, samples were diluted with RIPA and double the dilution buffer (100 mM Tris, pH 6.8, 6.0 M urea, 5% glycerol, 2% SDS, 0.01% bromophenol blue, 5% β-mercaptoethanol). After boiling (95 °C) for 5 min, samples were centrifuged for 1 min at 290 x *g* (Hettich Rotina 420 R) to collect the supernatant. For protein separation, 10 µL of each sample were transferred to 4-20% polyacrylamide precast gels (#4561095, BioRad) and a voltage of 150-180V) was applied for 80 min for electrophoresis in a tetra system^®^ (BioRad). For WB analysis, proteins were transferred to 0.2 µm PVDF membranes (trans-blot turbo #1704156, 2.5 A constant, up to 25 V, 7 min, BioRad). The membrane was blocked in blocking solution 3% BSA-PBST (137 mM NaCl, 5.4 mM KCl, 6.9 mM NaHCO_3_, pH 7.4, containing 0.5% Tween 20) for 1 h at RT and reacted with primary antibodies (anti-calprotectin S100A8, mouse, Origene BM4029 and anti-calprotectin S100A9, mouse, Origene BM4027), diluted 1:1000 in blocking solution, overnight at 4 °C. Following three washes for 10 min in TBS-T (150 mM NaCl, 50 mM Tris-HCL, pH 7.6, 0.5% Tween 20; all Sigma-Aldrich), blots were incubated in adequate secondary antibody solutions (goat anti-mouse IgG peroxidase conjugated, Pierce, #31430; goat anti-rabbit IgG peroxidase conjugated, Pierce, #31460; both 1:40000) for 30 min at RT. TBS-T-based washing was repeated. Signal detection was accomplished by an enhanced chemiluminescence detection system (Clarity Max Western ECL substrate, #1705062, BioRad) and recorded by a ChemiDOC Imager^®^ (BioRad). Protein masses were controlled by a protein ladder (PageRuler Plus Prestained Protein Ladder ~10–180 kDa, #26616, Thermo Fisher Scientific).

### 2.6 Assessment of CD15^+^, MPO- and citrullinated histone–positive cells in urine samples by flow cytometry

For the quantification of CD15^+^, MPO- and cit.H-positive cells by flow cytometry, urine samples were prepared as described by Lee et al. (29) with minor modifications. 1 mL of each urine sample was centrifuged at 211 x *g* for 5 min, the supernatant was discarded and the cell pellet suspended in 500 µL of PBS. This step was repeated twice before fixing the cells with PFA (4% final concentration) for 15 min at 4 °C. After fixation, the cells were washed thrice with 100 µL of PBS and then stored at -20 °C for further processing. All samples were processed in batches and not individually to minimize the inter-experiment variation. After thawing, the samples were washed (800 × *g* for 10 min at RT) and re-suspended in 200 µL PBS/1% BSA. Half of the sample was used for unstained controls and the remaining cells (100 µL) were subjected to a triple staining with primary antibodies recognizing MPO, cit.H and CD15 (**Table 2**). For staining, cells were incubated in 5 µL of Human BD Fc Block (BD Pharmingen Cat# 564220) for 10 min at RT. Then, 1 µg of each primary antibody was added and the samples were reacted for 60 min on ice, protected from light. After incubation, the samples were washed by adding 400 µL of PBS/1% BSA followed by a centrifugation step of 800 × *g* for 10 min. The cell pellets were resuspended in 250 µL of secondary antibody diluted in PBS (1:500) (**Table 2**) and incubated in the dark for 30 min at RT. Thereafter, cells were washed in 300 µL PBS/1% BSA (600 × *g* at RT). The cells were resuspended in 400 µL PBS/1% BSA. FCA was performed in a BD Accuri C6 plus^®^ (BD Biosciences, Heidelberg, Germany). A minimum of 200 µL per sample was analyzed under slow flow settings, irrespective of cell numbers. Gating was performed on single cells in the FL2 channel to identify CD15^+^ cells using the following strategy: Cells > Single cells > CD15^+^ (FL2) > CD15^+/^MPO^+^ (FL1)/cit.H (FL4). Cell analyses including assessment of CD15^+^ cell proportion were performed by the BD Accuri C6 plus^®^ analysis software module.

**Table 2:** Primary and secondary antibodies used for flow cytometry.

Antigen	Dilution	Company	Cat. no.	Origin	Secondary antibody
Histone H4 Arg3 citrullination (cit.H)	1:20	Millipore	07-596	rabbit	Alexa Fluor 647, goat anti-rabbit, Invitrogen, #A21244 1:500
MPO FITC-conjugated	1:100	biorbyt	orb16003	rabbit	-
CD15 PE-conjugated	1:20	BD Pharmingen	562371	mouse	-

### 2.7 Microbiome analysis

#### 2.7.1 Extraction of DNA for microbiome analysis

For bacterial nucleic acid extraction, 1 mL of urine was centrifuged at 16000 x *g* for 10 min. The cell pellets were lysed using a Vortex-Genie 2 (Thermo Fisher Scientific, Waltham, MA, USA) with 2 mL of BashingBeads (Zymo Research, Irvine, CA, USA) in accordance with the Optimised Lysis Protocol (Zymo Research, Irvine, CA, USA). DNA was then purified according to the manufacturer’s instructions using the ZymoBIOMICS DNA Miniprep Kit (Zymo Research, Irvine, CA, USA). All centrifugation steps were performed at 16000 x *g* unless otherwise specified in the instructions. Finally, DNA was eluted in 50 μL DNase/RNase-Free Water and quantified with the Quant-iT PicoGreen dsDNA Reagent Kit (Invitrogen, Life Technologies, Carlsbad, CA, USA).

#### 2.7.2 Amplification and library preparation of the V4 region of the 16S rRNA gene

To amplify the specific V4 region of the 16S rRNA gene, 10 pmol forward and reverse custom primers (Eurofins Scientific, Luxembourg City, Luxembourg) based on Caporaso et al. (2010) (30), Platinum SuperFi II PCR Master Mix (2x) (Thermo Fisher Scientific Inc., Waltham, MA, USA), and up to 20 ng in 10 μL of each extracted DNA sample were used in 10 μL. The V4 hypervariable region of the 16S rRNA gene was targeted due to its cost-efficiency and amplicon length of 291 bp (31) which enables almost complete overlap of sequenced reads, reducing error rates (30).

PCR amplification was performed following the manufacturer’s 3-step protocol, comprising 10 s of denaturation, 30 s of extension, and a total of 25 cycles. Amplified DNA was purified with 0.8x AMPure XP Beads (Beckman Coulter, Pasadena, CA, USA). To facilitate multiplex sequencing, 5 μL of each library was further amplified with a custom set of 10 μmol unique dual indices (Integrated DNA Technologies, Coralville, IA, USA) and NEBNext Ultra II Q5 Master Mix (New England Biolabs, Ipswich, MA, USA) according to the manufacturer’s protocol for NGS PCR. Following another purification using 0.8x AMPure XP Beads (Beckman Coulter, Pasadena, CA, USA), the libraries were quantified with the Quant-iT PicoGreen dsDNA Reagent Kit (Invitrogen, Life Technologies, Carlsbad, CA, USA). Libraries were then pooled in equimolar ratios and diluted to a final concentration of 4 nM.

#### 2.7.3 Short-read sequencing with Illumina MiSeq

The concentration of the library pool was measured with the Invitrogen Qubit 3 Fluorometer and a Qubit DNA High Sensitivity (HS) Kit (Thermo Fisher Scientific Inc., Waltham, MA, USA). Quality and sequence length distribution were assessed using the Bioanalyzer 2100 instrument with the DNA High Sensitivity Kit (Agilent, Santa Clara, CA, USA). Paired-end reads were sequenced on the MiSeq sequencing device using the MiSeq Reagent Nano Kit v2 (500 cycles) (Illumina, San Diego, CA, USA) and 20% of PhiX Control v3 (Illumina, San Diego, USA). The generated reads were image-processed, basecalled, and demultiplexed prior to analysis.

#### 2.7.4 Control of contaminations

To monitor potential contaminations or kit bias, and to verify that the implementation in the laboratory process was sufficient, several control samples were added at various steps of the workflow. To identify and eliminate bias caused by the extraction method, ZymoBIOMICS Gut Microbiome Standard (Zymo Research, Irvine, CA, USA) and kit isolation controls were isolated along with the rest of the samples. For insight into amplification bias and the processing of the isolated DNA, ZymoBIOMICS Microbial Community DNA Standard (Zymo Research, Irvine, CA, USA), as well as Ambion nuclease-free water (Life Technologies, Carlsbad, CA, USA), and water as a no-template control were added prior to amplification.

#### 2.7.5 Bioinformatics analysis of microbiome data

The 16S rRNA gene amplicon data were analysed as previously described in (32). Bar charts, boxplots, and PCoA results were visualized with the in-house software m3p.

### 2.8 Statistical analysis

Statistical significance was defined by a *p*-value ≤ 0.05. Normality of the data was assessed via the Shapiro-Wilk test. For IFM, ELISA and flow cytometry experiments, the *p*-values between groups were determined by non-parametric Kruskal-Wallis test followed by Dunn´s multiple comparison tests. Data were calculated as mean ± standard deviation (SD). All graphs and statistical analyses were performed using GraphPad Prism^®^ software (GraphPad Software Inc., V10.4.1; La Jolla, CA, USA).

The association between NETosis and UTI status was examined using two-way ANOVA with gender and UTI status as factors. *Post-hoc* comparisons were performed using Tukey’s test based on estimated marginal means. To adjust for potential confounders in patients with UTI, multivariable linear regression models were constructed including age, diabetes status, recurrent infection, and UTI diagnosis type as predictors. Model assumptions were assessed using Levene’s test, Shapiro-Wilk test, and visual inspection of residual plots. All analyses were conducted in jamovi (Version 2.6) with R (Version 4.4).

## 3 Results

### 3.1 Urine sample exclusion via Bilsen score-based evaluation

In total, 71 urine samples were collected and objectively classified according to the Bilsen Score (22) as described in section 2.2 (study design and sample collection). A detailed list of scores for all urine samples is provided in **Supplementary Table 1**. Samples scoring < 3 in UTI groups A or B, or > 0 in the control group, were excluded. Consequently, seven samples were removed from the study. Three female control samples (C-6, C-10, and C-15) scored above 0. In addition, one male control sample (C-18) was excluded due to the presence of sperm, which rendered it unsuitable for microscopic analysis. Further, one female UTI sample (A-7) and two male UTI samples (B-11 and B-18) were excluded due to the absence of leukocytes. As a result, the group sizes were revised: group A (women with UTI, *n* = 24), group B (men with UTI, *n* = 20), and group C (control group of healthy individuals of mixed gender, *n* = 20).

### 3.2 Live cell and IFM analyses unveil extracellular DNA in urine samples of UTI patients

**Figure 1:**
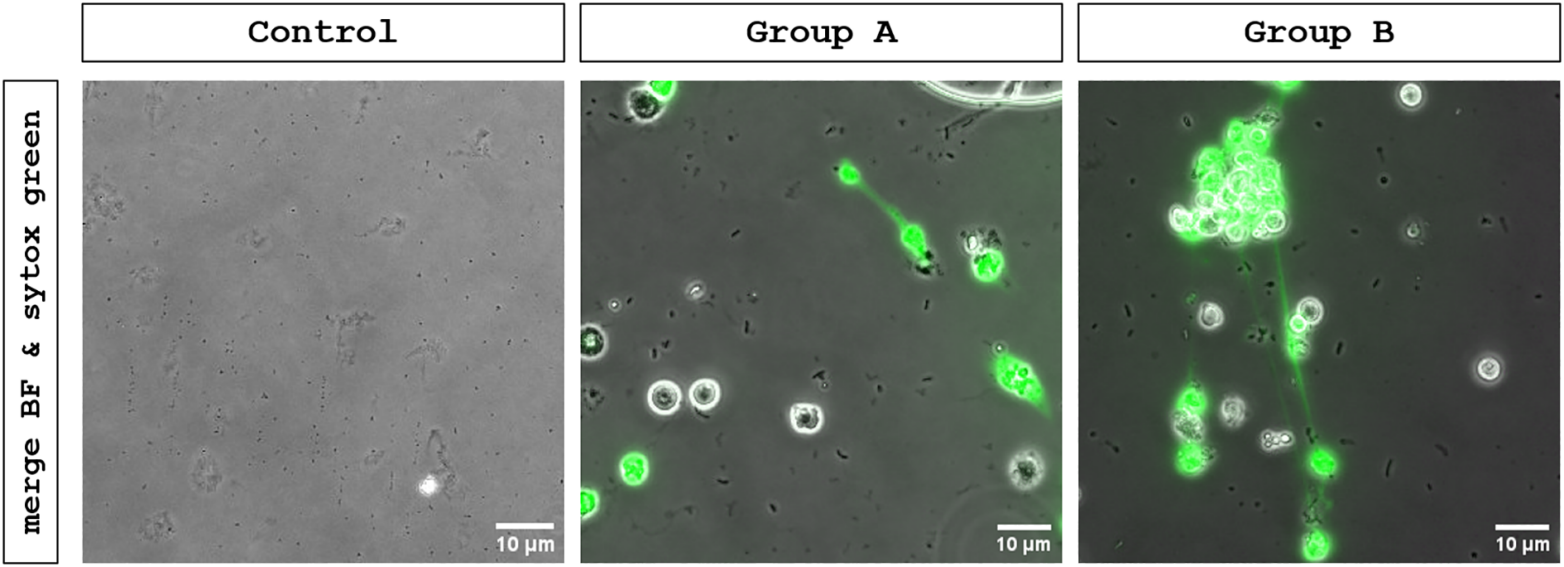
Live-cell fluorescence microscopic (LCFM) analysis of human urine samples for the detection of bacterial colonization, leukocyturia, cellular activity and NET release. LCFM analysis using Sytox Green (DNA staining) to visualize cellular structures, was performed on freshly collected urine samples across all groups. An inverted IX81 epifluorescence microscope equipped with an XM 10 digital camera (both Olympus) was used for image acquisition. Images show the merge of brightfield and DNA (green). Bacteria (indicated by a white arrow in Group B) and urinary NET- like structures were exclusively detected in UTI urines (Group A and B). Neither bacteria, cells, nor Sytox green fluorescence signals were observed in the negative controls (Control).

**Figure 2:**
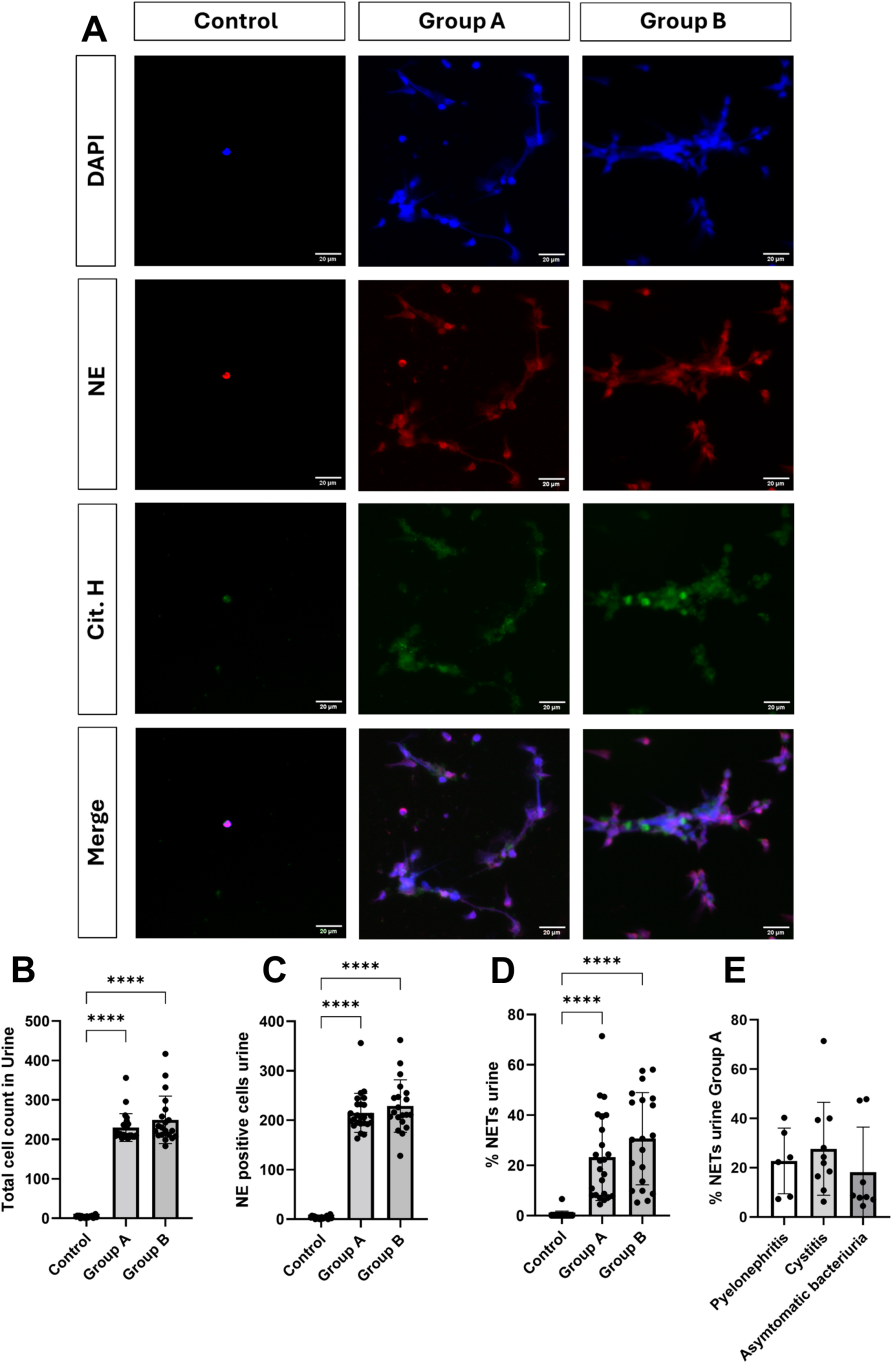
NET detection and quantification in urine by immunofluorescence microscopy (IFM). **(A)** NET structures were identified by the co-localization of DNA (blue), neutrophil elastase (NE; red) and citrullinated histones (green). Imaging was performed using a BZ-X800 microscope (Keyence), with images shown in individual channels and merged. In UTI urines (Group A and B) NETs were detected, while no NET formation was observed in healthy controls (Control). **(B–E)** NETs were quantified manually by microscopy in five randomly chosen fields per sample. Bar represents mean ± SD of each group. **(B)** Total cell counts were determined manually in the DAPI channel using the FIJI/Image J counting tool. **(C)** The proportion of human PMN in each sample was calculated by dividing the number of PMN in the NE channel by the total cell number in the DAPI channel. **(D)** The percentage of NETs was calculated as [number of NET-positive PMN/total PMN] x 100, and the average NET percentage was used for group comparison. **(E)** The percentage of NETs was calculated as in **(D)** but compared only between subgroups in Group **(A)** Total cell counts (B, DNA-positive; C, NE-positive cells), as well as the total percentage of NETs **(D)**, were significantly higher in UTI urine samples (Group A and B) compared to healthy controls (Control). **(E)** No significant difference was observed between UTI subgroups in Group A. ****p < 0.0001.

**Figure 3:**
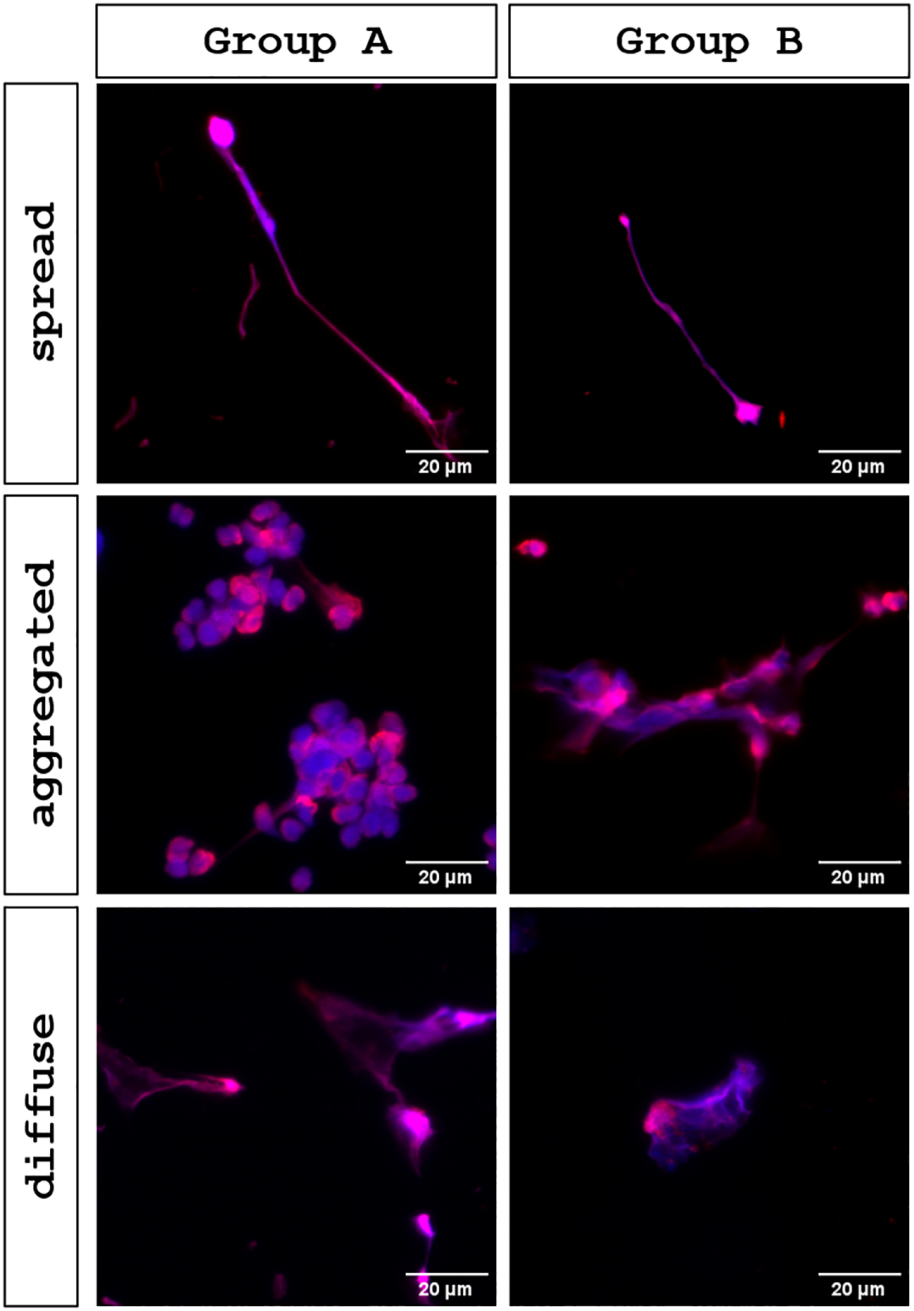
Presence of different NETs phenotypes (spread, aggregated and diffuse NETs) in UTI-derived urine samples. Urine samples of UTI groups (Group A and B) were examined for different NET phenotypes using immunofluorescence microscopy (IFM). Imaging was performed using a BZ-X800 microscope (Keyence), with images shown in in merge of extracellular DNA fibers (blue) decorated with neutrophil elastase (NE; red). In UTI urines (Group A and B), all different NETs phenotypes (spread, aggregated and diffuse NETs) were detectable.

To obtain an overview on fresh urine samples, samples were stained for DNA by Sytox Green^®^ and analyzed microscopically on the day of collection. This approach revealed bacterial colonization, leukocyturia and cellular activity in urine samples from patients with UTI, as shown in **Figure 1**. In these UTI samples, extracellular DNA filaments became visible, which appeared to be released from PMN, interacting with bacteria (**Figure 1**, white arrow). In contrast, urine samples originating from healthy patients (negative control) showed neither bacterial nor cellular elements and lacked Sytox Green^®^-based fluorescence signals (**Figure 1**). Since Sytox Green^®^ cannot distinguish between DNA sources, NETs must be double confirmed by an additional staining of typical NET markers. To detect NETs in urine, DNA fibers containing NET-associated proteins (i. e. cit.H, NE) were visualized by immunostaining. IFM analysis revealed extracellular DNA structures co-localizing with neutrophil elastase and citrullinated histones, fulfilling established criteria for NET identification (**Figure 2A**). Urines of UTI patients group A (female) and B (male) showed a significant presence of NETs, and this quantity varied between individuals, illustrated by representative IFM images and quantitative analyses (**Figures 2A–D**). Conversely, in group C (negative control) neither considerable numbers of PMN nor significant NET formation were detected (**Figures 2B–D**); occasionally, single inactivated PMN were found (please refer to **Figure 2A**). In contrast to the latter, an increase in PMN numbers was detected in urine samples originating from UTI patients (**Figure 2C**). Comparing NET percentages of individual groups, urine of UTI patients contained significant more NETs than urine originating from healthy donors (**Figure 2D**). Analyses revealed an average NET occurrence of 23.29% (SD +/- 16.89) in group A (females) and 30.63% (SD +/- 17.88) in group B (males). No significant difference of NET extrusion was observed between group A and B. As expected, the presence of PMN in urines of healthy individuals was low and NETs (0.32%, SD +/- 1.42) could hardly be detected (**Figure 2D**). Moreover, no significant differences in NET percentages were observed between group A-related subgroups: cystitis (27.72%, SD +/- 17.88), pyelonephritis (22.75%, SD +/- 12.91) and asymptomatic bacteriuria (18.17%, SD +/-17.14) (**Figure 2E**). Beyond quantitative differences, IFM analysis confirmed the presence of distinct NET phenotypes in UTI urine samples, i. e. *spr*NETs, *diff*NETs and *agg*NETs, as shown in **Figure 3**.

### 3.3 LL-37 and MPO concentrations are elevated in urine samples of UTI patients

**Figure 4:**
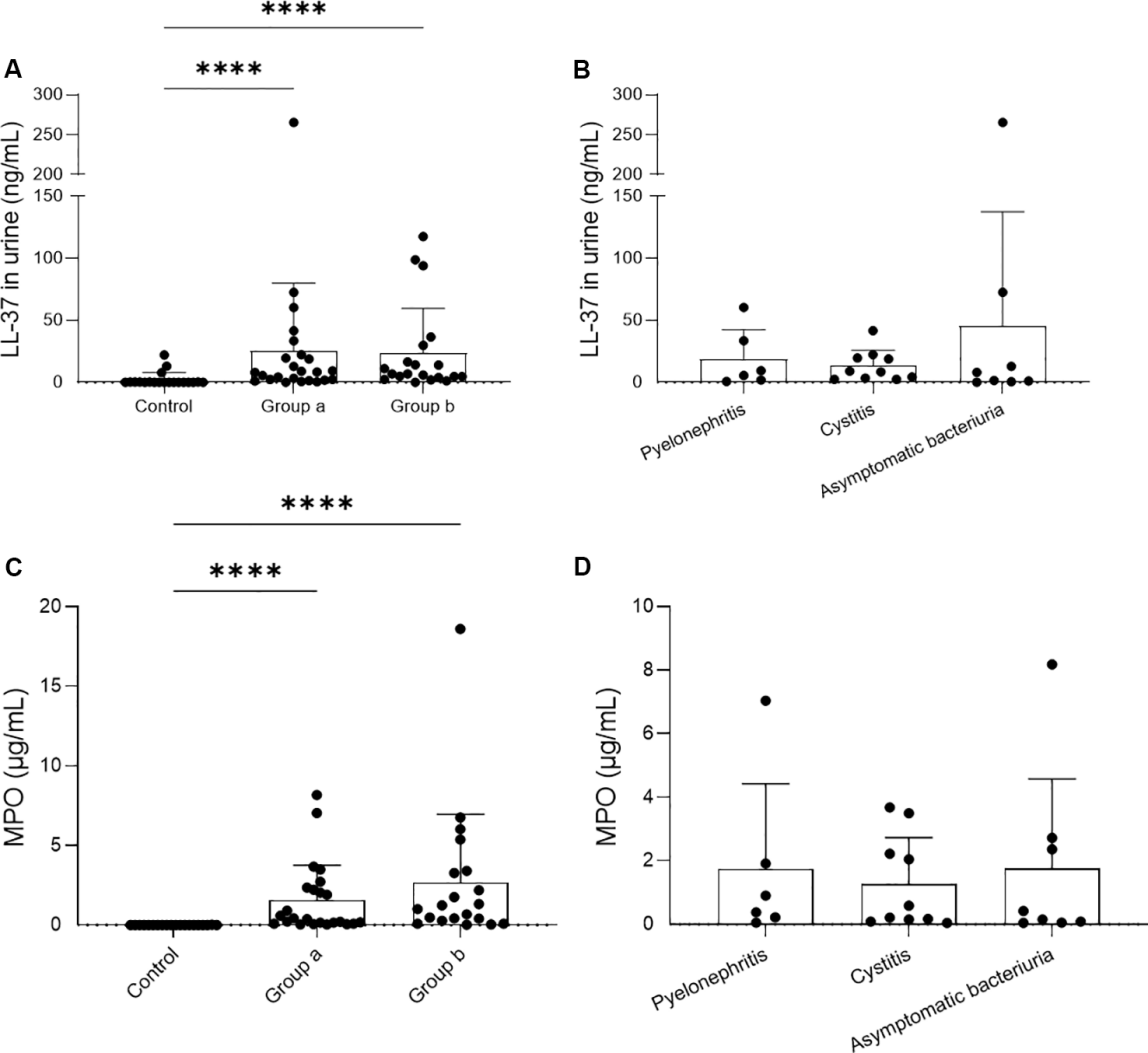
Detection of cathelicidin (LL-37) and myeloperoxidase (MPO) in human urine samples. Protein concentrations in human urine samples were detected by ELISA. The samples were analyzed in a microplate reader. Bar represents mean ± SD of each group. Protein levels were compared across groups **(A, C)** and subgroups (B, D). **(A, C)** LL-37 and MPO concentrations are higher in infected (Group A and B) than in healthy urines (Control). **(B, D)** No difference in protein concentrations was found when comparing the subgroups of Group A. ****p < 0.0001.

To investigate the concentration of NET-associated proteins LL-37 and MPO in urine, two ELISAs were performed. Consistent with the increased presence of PMN and NETs observed by immunofluorescence microscopy, LL-37 and MPO concentrations were significantly elevated in infected urines compared to controls, although showing high interindividual variation among UTI patients (**Figures 4A, C**). No quantitative differences were observed within the UTI group, neither between males and females (**Figures 4A, C**) nor among the subgroups within group A (**Figures 4B, D**).

### 3.4 Calprotectin and its subunits S100A8 and S100A9 are detectable in UTI urine probes

**Figure 5:**
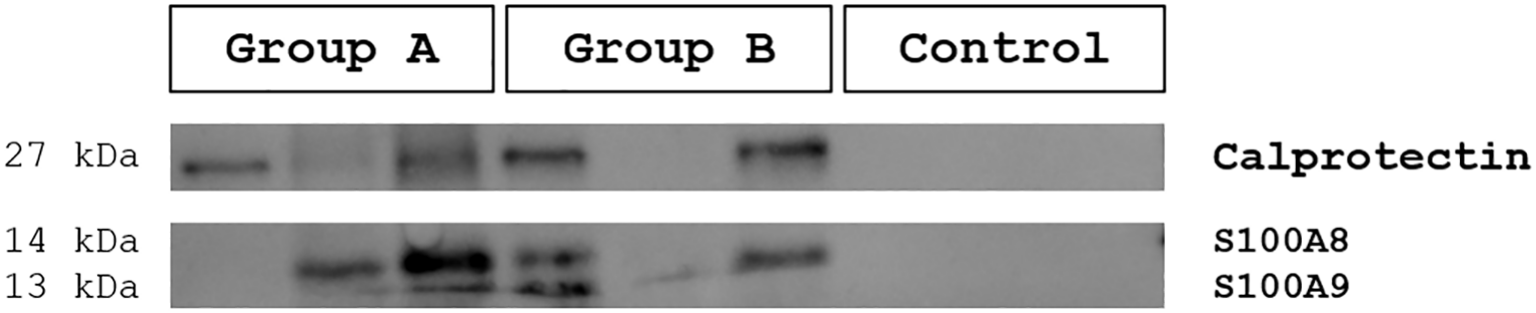
Detection of calprotectin and its subunits S100A9 and S100A8 in human urine samples via Western Blot (WB) analysis. Precast gels were loaded with each three samples per group (A = UTI in females, B = UTI in male, C = healthy control). Positive protein bands were visible at approximately 13 kDa (S100A8), 14 kDa (S100A9) and 27 kDa (Calprotectin). Calprotectin and its subunits S100A9 and S100A8 were detectable in infected urines (Group A, Group B) but absent in healthy patients (Control).

To assess the presence of the NET-associated protein calprotectin and its subunits S100A8 and S100A9 in urine, Western blot analysis was performed as a qualitative screening approach, thereby only estimating the presence or absence of the proteins. Therefore, three samples per group were controlled for the presence of related protein bands of approximately 11- (S100A8), 12.9/13.3- (S100A9) and 25 kDa (calprotectin) (**Figure 5**). Positive samples were counted and compared per (sub)group (**Table 3**). Calprotectin and its subunits were found in most urines from groups A and B [i. e. females with different UTI forms and males with biofilms (CA-UTI)] but was not present in any of the urine probes from healthy patients (negative control), as shown in **Figure 5**. A detailed overview on each sample can be found in the supplementary data [**Supplementary Figures 1**, **2**].

**Table 3:** Calprotectin presence or absence across urine (sub)groups.

Group	Subgroup	Calprotectin-positive samples
Control	–	0% (0/20)
Group A	all samples Pyelonephritis Cystitis Asymptomatic bacteriuria	62.5% (15/24) 66.6% (4/6) 70.0% (7/10) 50.0% (4/8)
Group B	–	70.0% (14/20)

### 3.5 UTI-derived urine samples show elevated levels of CD15^+^ cells

**Figure 6:**
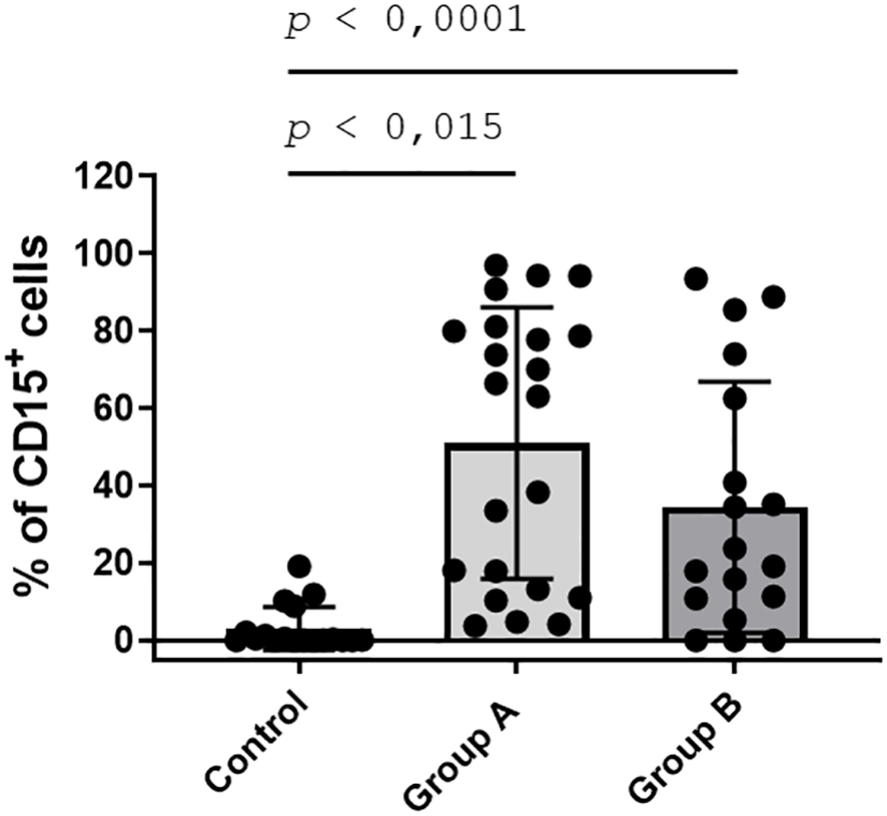
UTI-derived urine samples show elevated levels of CD15^+^ cells. The sediment of urine samples was stained for the myeloid marker CD15^+^ and analysed via flow cytometry. Results are expressed as percentage of CD15^+^ cells in the sample. Bar represents mean ± SD of each group. *P* values were calculated using a Kruskall-Wallis test of variance followed by a Dunnet´s multiple comparison test. CD15^+^ PMN numbers are increased in urines of UTI groups (Group A and B), but not in healthy urines (Control).

PMN heterogeneity under various maturation states can be observed through the differential expression of certain markers such as CD15. To determine CD15^+^ PMN in urine samples, flow cytometric analysis (FCA) was performed across all groups (**Figure 6**). FCA revealed significant differences between groups, with elevated percentages of CD15^+^ cells in UTI samples from groups A and B (females and males). In contrast, control samples hardly contained CD15^+^ cells (**Figure 6**). Group A exhibited a more homogeneous distribution of CD15^+^ cells compared to the variability observed in group B. In CD15^+^ cells, a high proportion (> 85%) proved positive for cit.H and MPO, indicating a NETotic phenotype.

### 3.6 Microbiome analysis

#### 3.6.1 Urine samples of UTI groups contain reduced microbial variability

**Figure 7:**
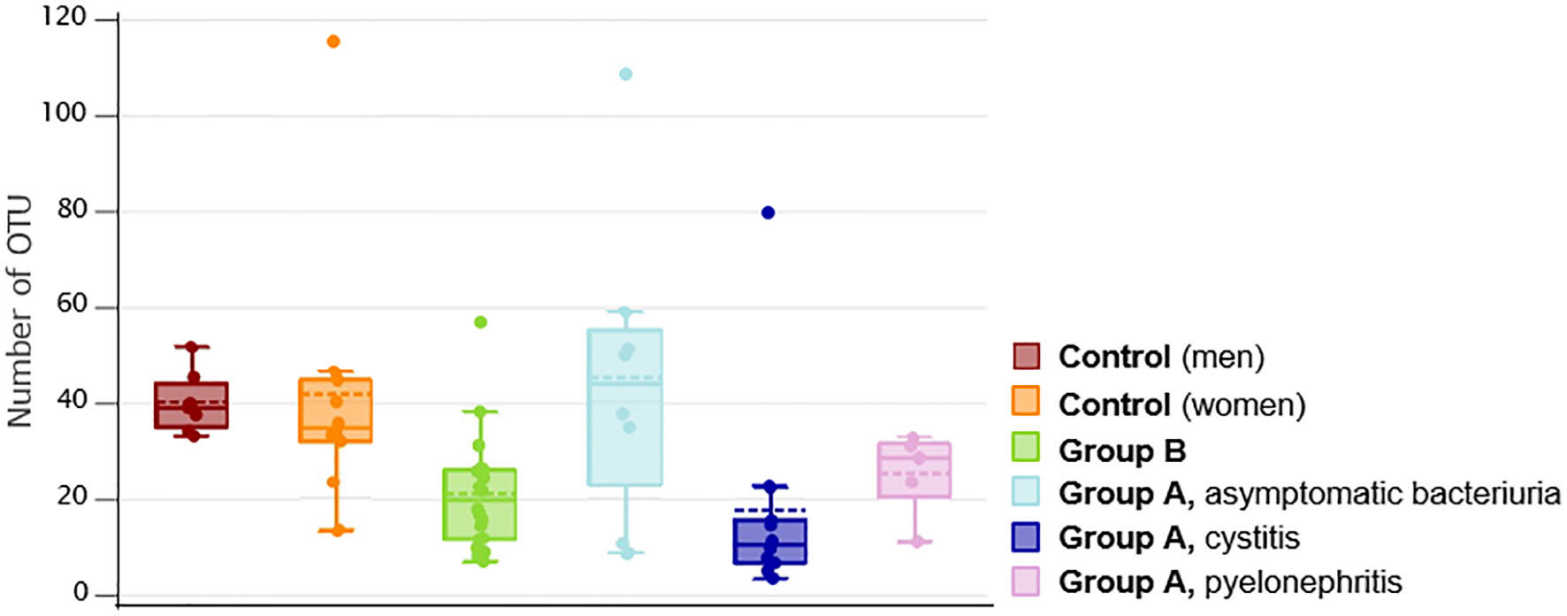
UTI urine samples show reduced OTU counts. The microbial community structure of all urine samples was estimated via 16S rRNA gene analysis and compared across groups and subgroups. The box plots visualize mean and median values, sample distribution, and potential outliers. A reduced number of Operational Taxonomic Units (OTUs), indicative of microbial genetic diversity, may suggest the dominance of a single pathogen. OTU numbers were lower in men with UTI (Group B) compared to healthy men (Control). Among UTI subgroups of Group A, pyelonephritis and cystitis showed notably reduced OTU counts, with cystitis exhibiting the lowest mean and median values. In contrast, the asymptomatic bacteriuria subgroup demonstrated a higher number of OTUs, closely resembling the levels observed in healthy individuals (Control).

16S rRNA gene analysis revealed the microbial community structure of urine samples. To illustrate potential differences in microbiome composition between UTI and healthy controls, samples were compared across groups and subgroups (**Figure 7**). Observed Richness (OR) represents the number of distinct Operational Taxonomic Units (OTUs) in a sample. Higher values indicate greater microbial diversity, whereas lower OTU counts may suggest the dominance of a single pathogen. The box plots visualize mean and median values, sample distribution, and potential outliers (**Figure 7**). The group A-related subgroups pyelonephritis (mean: 25.148/median: 28.23) and cystitis (mean: 17.606/median: 10.49) exhibited reduced OTU counts, with the cystitis subgroup displaying the lowest values in both mean and median. Similarly, men with bladder CA-UTI (Group B, mean: 20.997/median: 19.670) showed an overall lower OTU count compared to healthy controls. In contrast, the asymptomatic bacteriuria subgroup (mean: 45.675/median: 43.865) exhibited a higher OTU count, resembling the microbiome composition of healthy individuals (**Figure 7**). Control samples displayed a variability in OTU numbers: healthy men (mean: 39.975/median: 38.86) and healthy women (mean: 41.765/median: 34.66) showed distinct distributions, with some female samples exhibiting lower OTU counts. Outliers with particularly high OTU values were identified in the following (sub)groups: group A (cystitis and asymptomatic bacteriuria), group B and female control samples (**Figure 7**).

#### 3.6.2 Comparison of the top 50 different microbial families across (sub)groups

**Figure 8:**
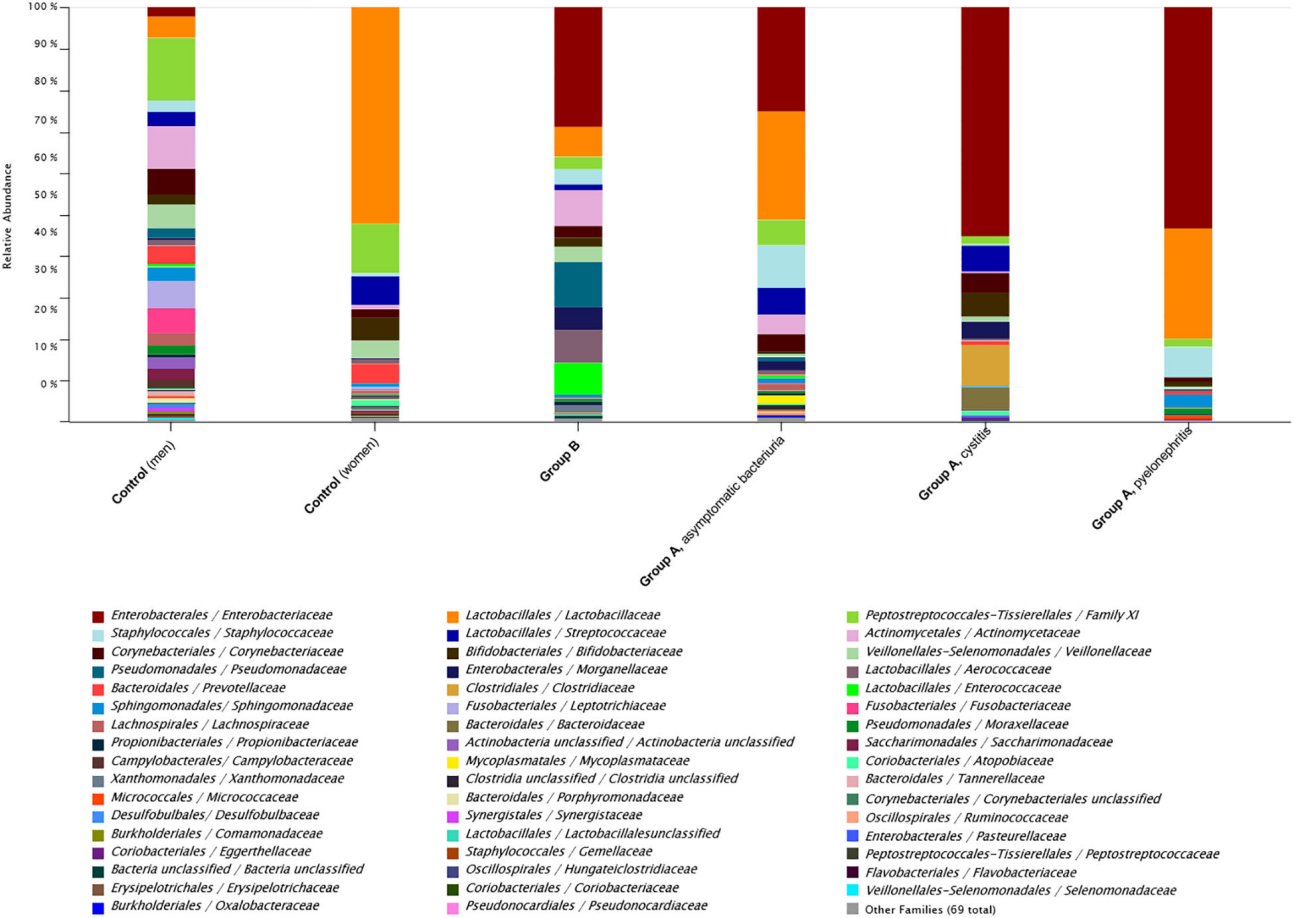
*Enterobacteriaceae* dominate the microbial composition of UTI groups. The microbial community structure of all urine samples was estimated via 16S rRNA gene analysis and compared across groups and subgroups. Taxonomic bar charts represent the relative abundance of the Top 50 different microbial families within the (sub)group. In UTI groups (Group A and B) *Enterobacteriaceae* dominate, whereas *Lactobacillaceae* were dominant in women, regardless of UTI status, with a notably higher proportion in healthy women (Control).

To illustrate the microbiome composition found in analysed urine samples, **Figure 8** lists the 50 most frequently detected bacterial families compared across (sub)groups. Predominant families in this comparison were the *Enterobacteriaceae* and *Lactobacillaceae*. The family *Enterobacteriaceae* was the most prevalent in the UTI groups (especially in cystitis and pyelonephritis) and was absent in healthy women and present only in minimal amounts in healthy men (**Figure 8**). *Lactobacillaceae* were dominant in women, regardless of UTI status, with a notably higher proportion in healthy women. Conversely, investigated men (both UTI and healthy ones) contained only small amounts of *Lactobacillaceae* (**Figure 8**).

#### 3.6.3 Clustering of urine samples based on microbiome similarity and NETosis in UTI patients

**Figure 9:**
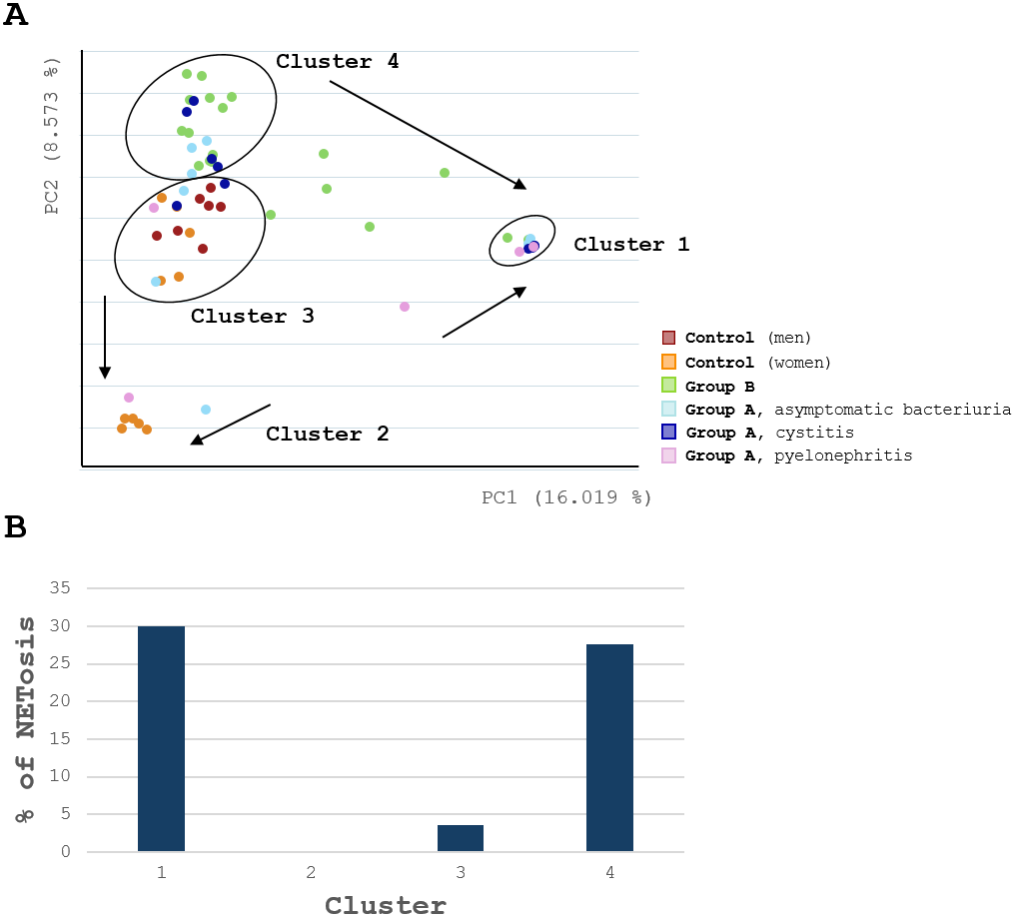
**(A)** Urine samples of (sub)groups cluster based on microbiome similarity. The microbial community structure of all urine samples was estimated via 16S rRNA gene analysis and compared across groups and subgroups. Urine samples clustered in Cluster 1–4 with outliers in between, showing tendencies toward specific cluster affiliations, as indicated by arrows. Healthy samples (Control) predominantly grouped on the left in Cluster 3. Samples with high *Lactobacillaceae* abundance were located in Clusters 2. UTI samples (Group A and B) primarily clustered in Cluster 4, while those dominated by *Enterobacteriaceae* formed a distinct Cluster 1. **(B)** NET formation is higher in Clusters with UTI samples. Urine samples were compared across Clusters, based on their average NETosis percentage from microscopic urine quantification and their microbiome composition. UTI Clusters 1 and 4 contain samples with higher NETotic potential, while control Clusters 2 and 3 containing mostly healthy urines, show little to no NETosis.

**Figure 9A** shows how urine samples clustered based on the similarity of their microbial composition derived from 16S rRNA gene analysis. Clusters 1 and 4 exclusively contained UTI samples from groups A and B. Cluster 1 was characterized by samples with a high proportion of *Enterobacteriaceae*, with an average proportion of 91.7%. Cluster 4 included UTI samples with other dominant microbes besides *Enterobacteriaceae*. Outliers showed a trend (indicated by arrows), suggesting a transition between the two UTI clusters, with samples exhibiting higher proportions of *Enterobacteriaceae* shifting towards cluster 1 (**Figure 9A**). Clusters 2 and 3 represented the control samples, with cluster 2 predominantly consisting of healthy women samples, with *Lactobacillaceae* as the dominating bacteria. Two outliers from group A, moving towards cluster 2 (indicated by arrows), also displayed a high proportion of *Lactobacillaceae* (**Figure 9A**). In cluster 3 a mixed composition of microbes was observed with no dominating bacterial species. Lower samples in this cluster tended to shift towards cluster 2, exhibiting higher proportions of *Lactobacillaceae*.

To further explore the relationship between urinary microbiome composition and NET formation, urine samples were compared across clusters based on their average NETosis percentage from microscopic urine quantification and their microbiome composition (**Figure 9B**). UTI clusters 1 and 4, with dominating pathogens, exhibited increased NET formation percentages (cluster 1: 29.99%, cluster 4: 27.65%). In contrast, clusters 2 and 3, which were primarily composed of control samples, displayed minimal to no NETotic activities (cluster 2: 0%, cluster 3: 3.63%).

We performed additional multivariable analyses adjusting for age, gender, diabetes status, and recurrent infection history. Two-way ANOVA confirmed a significant main effect of UTI status (*p*<.001) independent of gender (*p*=0.344), with no significant interaction (*p*=0.344). Linear regression models including age, diabetes, and recurrent infection as covariates revealed no significant associations of these variables with NETosis levels. The type of UTI diagnosis (cystitis, pyelonephritis, asymptomatic bacteriuria) also showed no significant effect (*p*=0.525).

## 4 Discussion

Cystitis is the most common UTI in humans (18, 21). This infection can ascend the urinary tract thereby extending to the renal pelvis, and potentially leading to severe conditions, such as pyelonephritis or urosepsis (18). Human UTI are primarily of bacterial origin, with *E.coli* as the most common urogenital pathogen (20, 21). A key defense mechanism of the innate immune system involves the formation of NETs which can entrap and kill microbial agents (3, 23, 25, 33). Consistently, the presence of NETs has been reported to occur in patients with acute and/or chronic urogenital tract infections of humans including epididymitis, vaginitis and polycystic ovary syndrome (34–36). Several studies suggest a link between NETs and UTI pathogenicity, which is not yet fully understood (23, 24). Elevated levels of NETs, exDNA and related proteins, such as LL37 and MPO, were detectable in UTI urines and associated with leukocyturia (23). Additionally, drugs like furosemide, which influence the kidney’s electrolyte balance and reduce NET formation, may exacerbate pyelonephritis (24).

The main objectives of this study were to clarify the relationship of NETosis in different UTI types with their underlying urinary microbiome, as well as the occurrence of urinary NETosis as a potential inflammatory biomarker in clinical human UTIs. In total, 71 patients were enrolled in this study and categorized into three cohorts: group A (women with cystitis, pyelonephritis, or asymptomatic bacteriuria); group B (men with CA-UTI) and group C (mixed gender control group). Seven samples had to be excluded retrospectively as described in section *3.1*, resulting in a final total of sixty-four samples (*n* = 64). NET-related experiments included the detection of extracellular DNA by live cell fluorescence microscopy and the detection of DNA-positive NET structures via colocalization analyses on DAPI, NE and cit.H, according to previous reports (37, 38). Manual quantification methods were chosen because semi-automated image analysis tools frequently failed to reliably identify individual NET structures, often missing NETs or incorrectly classifying multiple NETs as a single large structure. As a result, manual analysis provided more accurate and reproducible results (39). Along with assessing urine concentrations of the NET-associated proteins MPO and LL-37 (ELISA) (23), the presence of calprotectin and its S100A8/S100A9 subunits was analyzed by Western blotting (40). Finally, a quantification of CD15^+^ PMN was performed via FCA.

The different herein applied analyses resulted in a common core statement: regardless of the UTI type (classified by gender or specific UTI subgroups), urine samples from groups A and B contained high levels of CD15^+^ PMN, NETs and NET-derived proteins (i. e. NE, cit.H, MPO, LL-37 and calprotectin) in contrast to the control group (healthy patients). Of note, urinary calprotectin concentration was proposed as biomarker for UTI in adults and children under two years of age recently (16, 41). Additionally, all three different NET phenotypes were identified in UTI urines (8, 42). Nonetheless, significant differences were neither observed between the results of UTI groups A and B (female and male) nor between the subgroups within group A (pyelonephritis, cystitis, asymptomatic bacteriuria). Urinary NET percentages and protein concentrations exhibited high interindividual variations within the groups. The exact causes of this variability remain unclear, however, potential factors may include differences between patients, such as pre-existing medical conditions, varying levels of inflammation, individual microbiomes and/or hygiene practices. This phenomenon warrants further investigation with a larger sample size, as the smallest subgroup comprised only 6 patients, making it difficult to draw final conclusions. Nevertheless, when comparing UTI groups with the control group, it became evident that in the absence of infection, no PMN (or other leukocytes), bacteria, or proteins were detectable and, consequently, no urinary NETs were present.

To date, only limited literature is available on NETs in urine, however, some results of Stewart et al. (2024) (18) contrast current findings since they demonstrated the presence of PMN capable of forming NETs in urine from healthy donors and NET markers on urine-derived extracellular vesicles (EVs). NETs were composed of MPO-coated, cell-free DNA in healthy urine, suggesting that NETs are present in uninfected urinary tract forming part of a basal antimicrobial strategy. In our results, healthy urine samples contained neither PMN nor NETs, and only occasionally a single intact PMN was observed. Accordingly, extracellular DNA (exDNA) and NET-associated proteins like calprotectin and MPO were absent in control urines, while only three samples contained LL-37 at low level. The differences can be explained by the initial amount of urine since Stewart et al. (18), used 500 ml vs 10 ml in this study and differential centrifugation speeds and time. Of note, we correlated also the cell counts with NE presence as a marker of PMN. PMN present in healthy urine reported by Stewart et al. (18), shows less nuclear area and perimeter. This criterion was not considered at the time when the samples were analyzed. Additionally, the study by Stewart et al. (2024) (18) involved treating healthy urine samples with PMA, and other strong NETosis-inducing factors, allowing for the detection of structural components such as proteins. This approach was not employed in our study, and the absence of such a stimulus could be another potential explanatory factor for the differences observed. More research is needed to clarify this apparent divergence. The current data on NETosis are in line with previous UTI-related studies (16, 23, 43–46). As described in Krivošíková et al. (2023) (23) and consistent with the aim of this project, an increase of exDNA and antimicrobial proteins (MPO, LL-37), as these are structural components of NETs, was anticipated. Of note, MPO, implicated in pathogen killing and tissue damage, is known to play a pivotal role in chronic kidney disease (47) and was significantly elevated in UTI patients. Present MPO findings are in accordance with Ciragil et al. (2014) (44) detecting urine xanthine oxidase and MPO in the early phase of UTI. Further studies have shown a positive correlation between the MPO-to-creatinine ratio and leukocytosis in UTI patients (45). Moreover, as demonstrated by Chromek et al. (2006) (46) and in line with our results, low concentrations of LL-37 were detected in sterile urine, whereby these levels were significantly elevated during inflammation in cases of cystitis and pyelonephritis. The publication by Yu et al. (2017) (43) supports the current hypothesis of NETs playing a significant role in defense mechanisms in UTI by confirming early NET formation and associated proteins (i. e. MPO, citrullinated histones, NE) in PMN-rich urine samples. Comparable to our results, Przekora et al. (2024) (16) clearly showed that in UTI urines, calprotectin levels were significantly higher compared to febrile children without UTI or healthy children suggesting that this molecule may serve as a useful marker for febrile UTI in children. While these findings support the relevance of NETs and associated markers in UTI pathophysiology, Stoimeni et al. (2025) (48) concludes that their diagnostic utility is currently limited. This limitation arises mainly from methodological variability between studies and the absence of a standardized assay for NET detection (48). Differences between assays in sensitivity and specificity, together with technical variability, result in inconsistent findings across studies. As a result, further clinical validation and more standardized approaches to NET detection are needed before NETs can be used as reliable diagnostic biomarkers in routine practice (48). Overall, current data support the notion that PMN- and NET-derived markers can be useful to complement current standards on point-of-care tests. The final usefulness, however, will depend on not only the sensitivity and the time needed to obtain a result, but also on the associated costs.

The current microbiome analysis revealed distinct differences between UTI and control groups, suggesting urobiome dysbiosis, as shown in the aggregated evidence publication by Palumbo S et al., Urology 2025 (49). Firstly, urine microbiome sequencing data were examined for their microbial composition and grouped into OTU based on their sequence similarity (50). Samples from UTI groups exhibited lower OTU counts compared to healthy controls, supporting the idea that UTI are associated with a reduced microbial spectrum, consistent with the findings of Weng et al. (2023) (51) and Palumbo et al. (2025) (49). Former analysis similarly demonstrated reduced microbiota diversity and increased dominant bacteria in UTI patients versus controls (51). Control samples from both men and women generally displayed higher OTU counts, with exception of two outliers among healthy females, which exhibited lower OTU numbers. These samples were predominantly composed of *Lactobacillus* (52). As a common component of the vaginal microbiota (52), its strong presence may also result from contamination during urine collection. Another exception was noted in the asymptomatic bacteriuria subgroup of women (group A), which displayed higher OTU counts and a more diverse bacterial spectrum, resembling the microbiome composition of healthy individuals. This could be due to the absence of symptoms, suggesting a lack of pathogenic bacteria overgrowth and a more diverse bacterial community (53). Interestingly, this finding contrasts with the male patients in group B, which also remained asymptomatic, but wearing long-term bladder catheters (replaced every 4–6 weeks), leading to CA-UTI. Unlike asymptomatic women, these men exhibited low OTU counts with dominant bacterial families (*Enterobacteriaceae, Pseudomonadaceae*). Prolonged catheterization increases CA-UTI risk daily by 3-7% (54) and promotes inflammation, tissue damage and even the development of proliferative diseases (54, 55). The catheter microenvironment favors distinct mechanisms of bacterial colonization, namely the biofilm formation, thereby enhancing antibiotic resistance as previously reported (56). While *E. coli* dominates uncomplicated UTI, CA-UTI shows a more diverse pathogen spectrum, including also *Candida* spp., *Enterococcus* spp., and *Pseudomonas aeruginosa* facilitated by the catheter environment (54).

Secondly, top 50 analysis revealed two bacterial families predominantly shaping the microbiome composition across the study groups: *Enterobacteriaceae* and *Lactobacillaceae*. In controls*, Enterobacteriaceae* were absent (women) or rare (men) but highly prevalent in UTI cases. This family includes *E. coli*, the most common uropathogen (20), frequently causing human UTI. *Lactobacillaceae* were present in high proportions in healthy women. Several factors may contribute to this observation. *Lactobacillaceae*, as a common component of the female vaginal microbiome (53), was identified as the dominant genus in a healthy female urobiome (56). Higher levels in healthy women may reflect collection differences, as healthy urine was collected via midstream while UTI samples used sterile catheters, which reduce but do not fully prevent contamination. Several studies (51–53) have discussed the potential protective role of *Lactobacillaceae* as a ‘symbiont’ in the female urinary tract, by maintaining an acidic environment, inhibiting pathogens and preventing various urogenital diseases. Weng et al. (2023) (51) reported that UTI samples showed high *E. coli* and low *Lactobacillus* spp. levels. As both have been suggested as microbiota markers for UTI, elevated *Lactobacillus* in healthy women may be protective, whereas its reduction in UTI patients may allow pathogen dominance.

Further, urine samples formed distinct clusters, based on microbiome similarities. They were categorized into ‘healthy clusters’ and ‘UTI clusters’. Comparison of clusters with IFM-detected NET percentages showed higher NET values in UTI clusters. Healthy clusters displayed no NETs, except for a few outliers from UTI samples, explaining slightly elevated values. An *Enterobacteriaceae*-dominated UTI cluster exhibited the highest NET percentage (29.99%), slightly exceeding that of a mixed-pathogen UTI cluster (27.65%). Our findings of the multivariable analysis indicate that the observed association between UTI and NETosis is robust to adjustment for the major clinical confounders available in our dataset. However, we acknowledge that the limited sample size restricts our ability to detect modest confounding effects or interactions. These results highlight the need for further research into how different bacterial species influence NET formation, immune evasion, and pro-inflammatory responses. Understanding these complex mechanisms could contribute to the development of targeted therapeutic strategies for UTI patients, although the polymicrobial nature of many complicated or recurrent UTIs poses a challenge. Such an approach may help determine whether specific uropathogens either enhance or inhibit NETosis. A study by Ou et al. (2021) (25) demonstrated that *E. coli* suppresses NET formation through TcpC, to degrade PAD4, a key enzyme in NET formation, thereby inhibiting NET-mediated bacterial clearance and evading host immunity (25).

This study had several limitations in material acquisition when working with human body fluids. Urine and blood samples were collected from UTI patients at the UKGM Giessen, Germany. Patients on prior antibiotic treatment were not included to avoid confounding effects on UTI innate defense mechanisms. This proved to be challenging, as UTI patients frequently had already received antibiotics from general practitioners before UKGM hospitalization. Additionally, late-night samples were unusable due to prolonged storage before processing. Written consent was required, and some patients or guardians declined participation. A further limitation is that in this pilot study a relatively small sample size was included, especially in the clinical UTI subgroups, which warrants further studies with larger samples sizes.

To conclude, this pilot study demonstrates the presence of PMN-derived NETs in urine samples of human UTI patients but showing high interindividual variations. In all UTI types (regardless of gender or subgroups), NETs of varying phenotypes and associated proteins were detected at higher concentrations than in healthy controls, improving our understanding in one of the most used clinical point-of-care tests. UTI samples exhibited reduced microbiome diversity compared to healthy controls, characterized by the predominance of infection causing pathogens. *Enterobacteriaceae* represented the largest proportion of uropathogenic bacteria in these samples.

## Data Availability

The datasets presented in this study can be found in online repositories. The names of the repository/repositories and accession number(s) can be found below: https://www.ncbi.nlm.nih.gov/, PRJNA1358065.
