# Supplementary material for: Presence of neutrophil extracellular traps (NETs) in different types of human urinary tract infections (UTI). A pilot study
Source: Front Immunol. 2026 Mar 16;17:1745166. doi: 10.3389/fimmu.2026.1745166 (PMC13033559; doi:10.3389/fimmu.2026.1745166)
Supplement: Supplementary file 1 [file DataSheet1.pdf]

Supplementary Material

1 Supplementary Figures and Tables

1.1 Supplementary tables

Supplementary table I: Patients probability for UTI using the Consensus Score of Bilsen et al. (Bilsen et al. 2024)

*marked patients were removed from further analysis for not meeting inclusion or meeting exclusion criteria.

group	patient number	symptoms	systemic criteria	pyuria	culture results	score
**A**	A-1	3	2	3	3	11, definite UTI
	A-2	3	2	3	1	9, definite UTI
	A-3	4	0	3	1	8, definite UTI
	A-4	3	2	3	3	11, definite UTI
	A-5	3	0	3	3	9, definite UTI
	A-6	2	0	3	2	7, probable UTI
	A-7*	4	0	0	1	5, possible UTI
	A-8	4	0	3	1	8, definite UTI
	A-9	3	0	3	3	9, definite UTI
	A-10	0	0	3	3	6, probable UTI
	A-11	0	0	3	1	4, possible UTI
	A-12	4	0	3	1	8, definite UTI
	A-13	0	0	3	1	4, possible UTI
	A-14	3	0	3	1	7, probable UTI
	A-15	0	0	2	1	3, possible UTI
	A-16	0	0	3	3	6, probable UTI
	A-17	3	0	3	3	9, definite UTI
	A-18	3	0	3	1	7, probable UTI
	A-19	0	0	3	1	4, possible UTI
	A-20	3	2	3	3	11, definite UTI
	A-21	4	0	3	2	9, definite UTI

	A-22	0	0	3	3	6, probable UTI
	A-23	0	0	3	1	4, possible UTI
	A-24	3	2	2	1	8, definite UTI
	A-25	3	2	3	3	11, definite UTI
**B**	B-1	0	0	3	3	6, probable UTI
	B-2	0	0	3	3	6, probable UTI
	B-3	0	0	3	3	6, probable UTI
	B-4	0	0	3	3	6, probable UTI
	B-5	0	0	2	3	5, probable UTI
	B-6	0	0	3	3	6, probable UTI
	B-7	3	2	3	1	9, definite UTI
	B-8	0	0	3	3	6, probable UTI
	B-9	0	0	3	3	6, probable UTI
	B-10	0	0	3	3	6, probable UTI
	B-11*	0	0	0	3	3, possible UTI
	B-12	0	0	2	1	3, possible UTI
	B-13	0	0	2	3	5, probable UTI
	B-14	0	0	3	3	6, probable UTI
	B-15	0	0	3	1	4, possible UTI
	B-16	0	0	3	1	4, possible UTI
	B-17	0	0	3	3	6, probable UTI
	B-18*	0	0	0	1	1, no UTI
	B-19	0	0	3	3	6, probable UTI
	B-20	0	0	3	3	6, probable UTI
	B-21	0	0	3	3	6, probable UTI
	B-22	0	0	3	1	4, possible UTI
**Control**	C-1	0	0	0	0	0, no UTI
	C-2	0	0	0	0	0, no UTI
	C-3	0	0	0	0	0, no UTI
	C-4	0	0	0	0	0, no UTI
	C-5	0	0	0	0	0, no UTI
	C-6*	0	0	0	1	1, no UTI

	C-7	0	0	0	0	0, no UTI
	C-8	0	0	0	0	0, no UTI
	C-9	0	0	0	0	0, no UTI
	C-10*	0	0	0	1	0, no UTI
	C-11	0	0	0	0	0, no UTI
	C-12	0	0	0	0	0, no UTI
	C-13	0	0	0	0	0, no UTI
	C-15*	0	0	2	0	2, no UTI
	C-16	0	0	0	0	0, no UTI
	C-17	0	0	0	0	0, no UTI
	C-18*	0	0	0	0	0, no UTI
	C-19	0	0	0	0	0, no UTI
	C-20	0	0	0	0	0, no UTI
	C-21	0	0	0	0	0, no UTI
	C-22	0	0	0	0	0, no UTI
	C-23	0	0	0	0	0, no UTI
	C-24	0	0	0	0	0, no UTI
	C-25	0	0	0	0	0, no UTI

1.2 Supplementary figure captions

Supplementary figure 1: Exemplary gel electrophoresis of urine samples. Precast gels were loaded with each three samples per group (A= UTI in females, B = UTI in male, C =negative control). The positive control consisted of isolated blood PMN. As reference, the protein ladder with the corresponding protein sizes in kDa is shown on the left. White arrows point the bands that detect calprotectin at approx. 25 kDa (upper arrow) and its subunits S100A8 approx. 11 kDa and S100A9 approx. 13 kDa (lower arrows).

Supplementary figure 2: All sample loadings and results. All gels were loaded with each three samples per group (A = UTI in females (orange), B = UTI in male (blue), C = negative control). Positive protein bands were visible at 12-14 kDa for subunits and 27-35 kDa for Calprotectin.
