# Supplementary figures and images for: Presence of neutrophil extracellular traps (NETs) in different types of human urinary tract infections (UTI). A pilot study
Source: Front Immunol. 2026 Mar 16;17:1745166. doi: 10.3389/fimmu.2026.1745166 (PMC13033559; doi:10.3389/fimmu.2026.1745166)

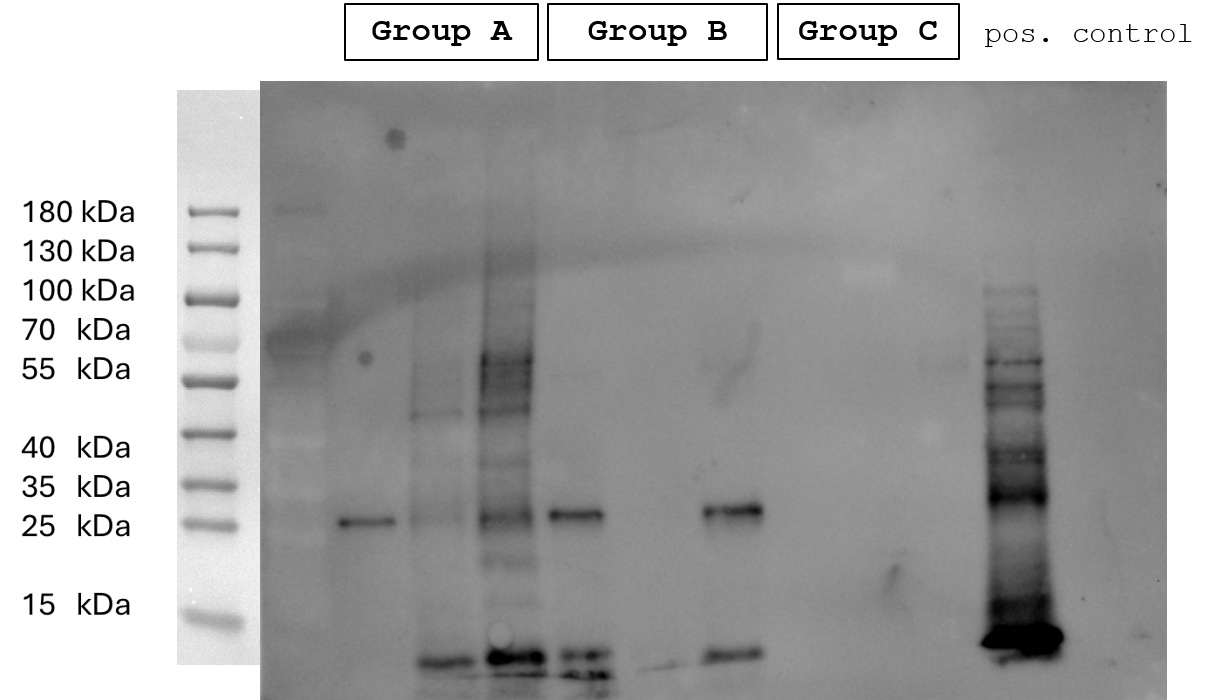

Supplement: Supplementary file 2 [file Image1.tif]

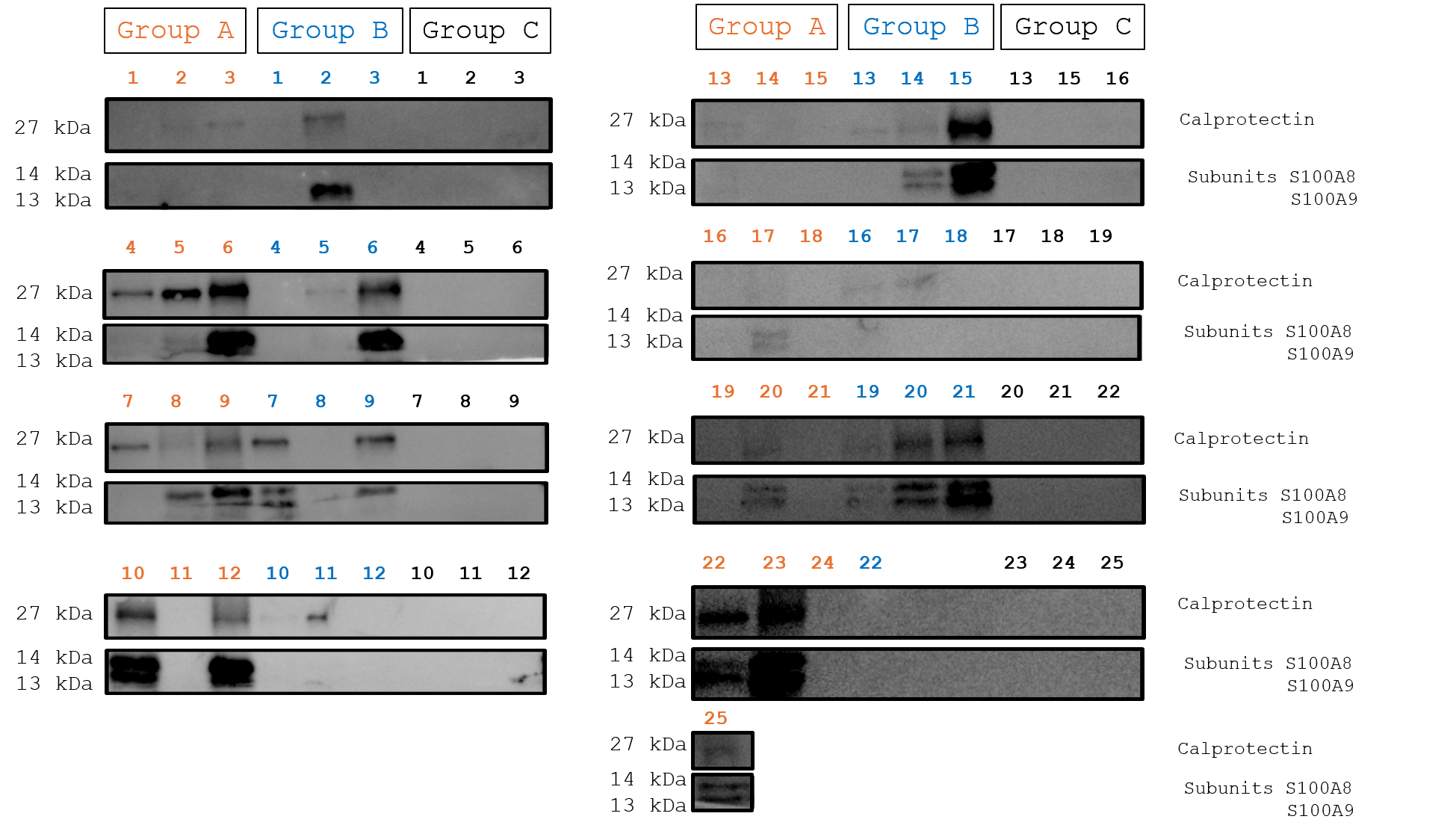

Supplement: Supplementary file 3 [file Image2.tif]
